# Growth-Promoting Potential of *Bacillus subtilis* GMG1288 in Wheat Under Abiotic Stress: Effects of Application Method and Timing

**DOI:** 10.3390/plants15182806

**Published:** 2026-09-13

**Authors:** Ekaterina Alexeevna Sokolova, Evgeniya Vladimirovna Chumanova, Irina Nikolaevna Tromenschleger, Elena Nikolaevna Voronina

**Affiliations:** Knorre Institute of Chemical Biology and Fundamental Medicine, Siberian Branch of the Russian Academy of Sciences, Novosibirsk 630090, Russia

**Keywords:** *Bacillus subtilis*, PGPB, abiotic stress, application method, application timing, salinity, osmotic stress, heavy metals

## Abstract

*Bacillus subtilis* is one of the most widely used plant growth-promoting bacteria (PGPB), but the optimal method and timing of its application under abiotic stress have not been systematically compared across different stress types within a single experimental framework. Here, we evaluated the effect of *B. subtilis* strain GMG1288 on spring wheat (*Triticum aestivum* L.) under three abiotic stresses—soil salinity, PEG-induced osmotic stress, and heavy metal contamination—comparing 13 application variants (seed inoculation, and single or multiple watering with the bacterial suspension) applied at different time points in a 6-week pot experiment, alongside in vitro characterization of strain GMG1288. Two-way ANOVA showed that stress type was the dominant source of variability in wheat growth parameters, while application method and timing were significant but weaker, stress-dependent modifiers. Under salinity, seed inoculation was the key determinant of root mass gain; under osmotic stress, application timing was decisive, with efficacy confined to a narrow window at stress onset (days 14–21); under heavy metal stress, only repeated watering produced a consistent effect, while single applications and inoculation showed no reliable benefit. These results indicate that the effective application regimen for *B. subtilis* GMG1288 is stress-specific rather than universal, and suggest that application strategies for PGPB-based biological products should be guided by strain-specific phenotypic activity profiles rather than by generic method/timing schemes.

## 1. Introduction

Wheat (*Triticum aestivum* L.) accounts for about 20% of the world’s calorie and protein consumption, serving as the staple food for more than 35% of the planet’s population [1]. However, the crop remains highly vulnerable to abiotic stresses: drought, high salinity, temperature extremes, and the toxicity of heavy metals are key factors that reduce yield by disrupting the physiological and biochemical pathways of plant development. Attempts to reduce crop losses by increasing the doses of mineral fertilizers and chemical ameliorants produce only limited results: they do not eliminate the osmotic and ionic components of stress, and the uncontrolled use of agrochemicals leads to the degradation of soil biotic communities and widespread environmental pollution, and remains economically unfeasible for a significant portion of producers in arid regions [2]. Against this backdrop, the use of plant growth-promoting bacteria (PGPB) is considered an environmentally safe and sustainable alternative to agrochemicals, aimed directly at increasing stress resistance through the modulation of hormonal balance, osmoprotection, and improved mineral nutrition.

Among microbial preparations used in crop production, *Bacillus subtilis* occupies a prominent position. Preparations based on this bacterium dominate the market for biocontrol agents and have proven effective in controlling major plant diseases [3]. The wide use of *B. subtilis*-based products can be attributed to a combination of properties, including the ability to form stable endospores, which ensures the long-term stability of the preparation and a long shelf life. Additionally, it produces a wide range of antimicrobial metabolites, such as surfactin, fengycin, and iturin, which contribute to its effectiveness [4]. Direct mechanisms of *B. subtilis* action include the synthesis of phytohormones that promote root elongation and stress resistance. It also solubilizes inorganic phosphate and produces siderophores and enzymes that mobilize nutrients. Indirect mechanisms involve suppressing phytopathogens and inducing systemic plant resistance. Under abiotic stress, the protective effect is achieved through the regulation of the plant’s hormonal balance, osmoprotection, and the colonization of the root system—a process that critically depends on biofilm formation and “swarming” motility [5,6]. For wheat under salt stress, the growth-promoting effect of *B. subtilis* on root length has been shown to increase in proportion to salinity intensity, accompanied by bacterial colonization of roots, stems, and leaves, as confirmed by scanning electron microscopy (SEM) and polymerase chain reaction (PCR) [7].

Meanwhile, the choice of when to apply PGPB has a fundamental biological basis. The effectiveness of PGPB depends on the ability of the bacteria to reach the root system and colonize it. This process fundamentally differs depending on the type of stress factor. In the case of soil salinity, high concentrations of sodium ions inhibit the mobility of *Bacillus* by suppressing the expression of chemotaxis and flagellar genes [8]. This is expected to reduce the likelihood of successful colonization of roots when applying irrigation in saline soils. When soils are contaminated with heavy metals such as copper and zinc, these ions selectively disrupt the expression of genes encoding extracellular matrix components without affecting planktonic cell growth [9]. This means that they do not affect the survival of bacteria, but rather their ability to attach to roots. Under osmotic stress, the accumulation of ABA and the decrease in IAA in the host plant can block the growth-stimulating response, regardless of the method of application [10]. These mechanisms have been described in isolated studies, usually within the context of a specific stress condition and specific application method. However, their combined effect on the efficacy of different application regimes has not been experimentally tested.

Nevertheless, there are very few comparative studies in the literature that systematically evaluate different application methods (seed inoculation, single and multiple irrigation with the suspension), and different time periods, under various types of abiotic stress, within a single experimental design. This lack of research prevents us from answering a fundamental practical question: Is there a universally optimal regime for using *Bacillus subtilis*, or does the optimal regime depend on the type of stress, and if so, what biological mechanisms underlie this phenomenon?

*Bacillus subtilis* strain GMG1288, obtained from the Center for Applied Microbiology at the Knorre Institute of Chemical Biology and Fundamental Medicine SB RAS (http://www.niboch.nsc.ru/doku.php/ru/structure/ckp-csm, (accessed on 19 August 2026)), served as the object of this study. This strain was chosen based on its resistance to abiotic stresses and its ability to produce plant growth-promoting substances in vitro. These characteristics make it a suitable candidate for a systematic evaluation of its effectiveness under multiple abiotic stress conditions.

The aim of this study was to determine the optimal method of applying *Bacillus subtilis* GMG1288 in order to enhance the growth parameters of wheat (*Triticum aestivum* L.) under different types of abiotic stress, such as salinity, osmotic stress, and heavy metal pollution. The central hypothesis was that the effectiveness of the application method and timing depends on the specific type of stress, as different stressors affect the key stages of interaction between the bacterium and plant in different ways, including mobility, biofilm formation, and host hormonal response. To achieve this aim, the following tasks were addressed: (1) to compare thirteen methods of applying *B. subtilis* GMG1288—seed inoculation, single and multiple irrigation with the suspension at different times—under four conditions of abiotic stress in a pot experiment; (2) to assess the relative contribution of the application method and timing, as well as their interaction with the type of stress, to the variability in wheat growth parameters.

## 2. Results

### 2.1. Phenotypic Characteristics of Bacillus subtilis GMG1288: Rationale for Selecting the Strain

The choice of the *Bacillus subtilis* GMG1288 strain for further experiments was justified based on its phenotypic resistance to abiotic stress in vitro and its production of metabolites that are key to growth-stimulating activity. The strain’s growth rate was assessed at increasing concentrations of osmotic agents (PEG), NaCl, and heavy metal ions. Additionally, the production of traits that are significant for plant growth promotion (PGP) activity was measured (Table 1).

The growth of *B. subtilis* GMG1288 was recorded across a range of PEG concentrations, including the maximum concentration of 15%, which corresponds to the criterion for osmotic stress resistance adopted within the strain collection (growth > 40% at 15% PEG). When NaCl was added, positive growth was maintained up to a concentration of 10% NaCl (53.32% ± 8.52%). In the presence of heavy metals (1000 mg/L, corresponding to 15.74 mM Cu^2+^, 8.90 mM Cd^2+^, 15.29 mM Zn^2+^, and 4.83 mM Pb^2+^), the strain showed no significant difference in growth compared to the control when Cu was added (121.93 ± 20.20%). Cd and Zn at the same concentration almost completely inhibited growth, with mean values of 0.84% ± 0.85% and 15.29% ± 9.96%, respectively.

Among the functional characteristics, phosphate mobilization is the most prominent (277.45 ± 46.60 μg/mL), which may underlie the growth-promoting effect under stress conditions, as phosphate availability in the soil decreases during both drought (due to limited diffusion in drying soil) and salinization (due to ion competition and precipitation). High production of proline (74.72 ± 2.94 μg/mL) primarily indicates the strain’s own osmotolerance—its ability to maintain metabolic activity under osmotically unfavorable conditions in the rhizosphere—which may further contribute to colonization and survival of the bacterium under stress. In contrast, production of IAA, ammonium, salicylic acid, and siderophores was not detected, suggesting that classical auxin- and siderophore-mediated mechanisms are not significant contributors in this strain.

Thus, the strain was selected for the pot experiment based on its resistance to salt and osmotic stress, including its capacity for osmotic adjustment via proline accumulation. In the following sections, we examine whether this phenotypic potential is expressed at the whole-plant level under different types of abiotic stress and with different methods and timings of biological product application.

### 2.2. The Overall Structure of Variability in Growth Indicators: The Relationship Between the Contribution of Stress and the Method and Timing of Application

Before conducting a detailed analysis of each stress factor, we assessed the proportion of variability in the growth and weight indicators of wheat that can be explained by the type of abiotic stress, the method of applying *Bacillus subtilis* GMG1288 (seed inoculation with or without additional watering; single watering without inoculation; multiple waterings without inoculation), and the timing of application (on days 0, 14, 21, or 28 after planting).

It should be noted that stress exposure began on day 15, reached its maximum on day 17, and was maintained until the end of the experiment. Specifically, administration on day 14 corresponded to the addition of the biological product immediately prior to the onset of stress, while administration on day 21 occurred 3 days after the peak of the stress load. To visualize the overall data structure, a principal component analysis was performed on the group means for all measured indicators (height, fresh and dry weight of leaves, roots, and the entire plant), with *n* = 52 (13 application methods × 4 conditions). Additionally, a two-factor analysis of variance (application method × stress and application time × stress) was conducted for statistical verification (Table 2).

**Figure 1:**
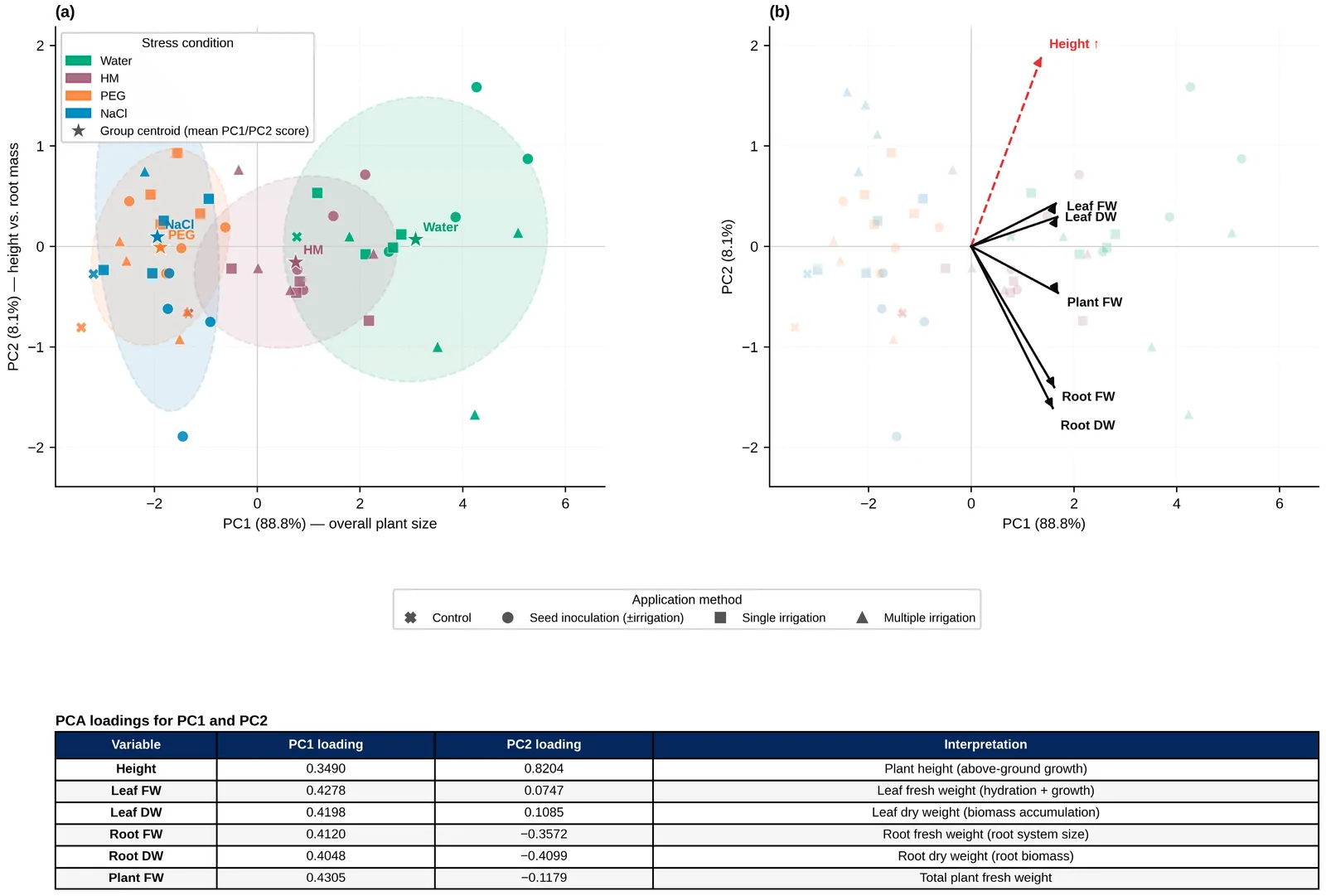
PCA. (**a**) PCA-biplot on group averages (13 stress options × 4): the dots are colored according to the type of stress; the shape of the marker is the method of application; (**b**) PCA-biplot of variable load.

The first principal component accounted for 90.2% of the total variance and combined all the measured indicators with similar loadings (0.32–0.40) (Figure 1). This component reflected the overall size of the plant and clearly separated the variants by type of stress. No visual separation between application groups was observed in the PCA, as their contribution to total variance was markedly less than that of stress. The second component (7.0%) contrasted shoot height (load +0.83) with root mass (–0.33…–0.38), which could be interpreted as an axis of biomass redistribution between the above-ground and root parts.

A two-factor ANOVA confirmed that the type of stress was the dominant source of variability across all indicators, with partial η^2^ values ranging from 0.51 for height to 0.77 for total plant mass (*p* < 0.001 everywhere). The application method, on the other hand, was a statistically significant factor, but its effect was considerably weaker (partial η^2^ = 0.10–0.13 for leaf and total plant mass). For height and root mass, the effect of application method was not significant or only borderline significant. In all cases, the “application method × stress” interaction was statistically significant, meaning that the effectiveness of the application method varied depending on the type of stress. The application period (day 0/14/21/28) made a significant contribution to the variability, regardless of stress (partial η^2^ = 0.15–0.21, *p* = 0.001–0.013). The “period × stress” interaction was not significant (*p* > 0.06), indicating that the effect of the application period was consistent across all types of stress.

The observed significant dependence of the effect of the application method on the type of stress (interaction method × stress) indicated that there is no single “best” method—its effectiveness must be evaluated separately for each stress condition. The results below are therefore presented accordingly.

### 2.3. Growth and Weight Indicators of Wheat Under Abiotic Stress Conditions in a Growing Experiment

#### 2.3.1. Heavy Metals

**Figure 2:**
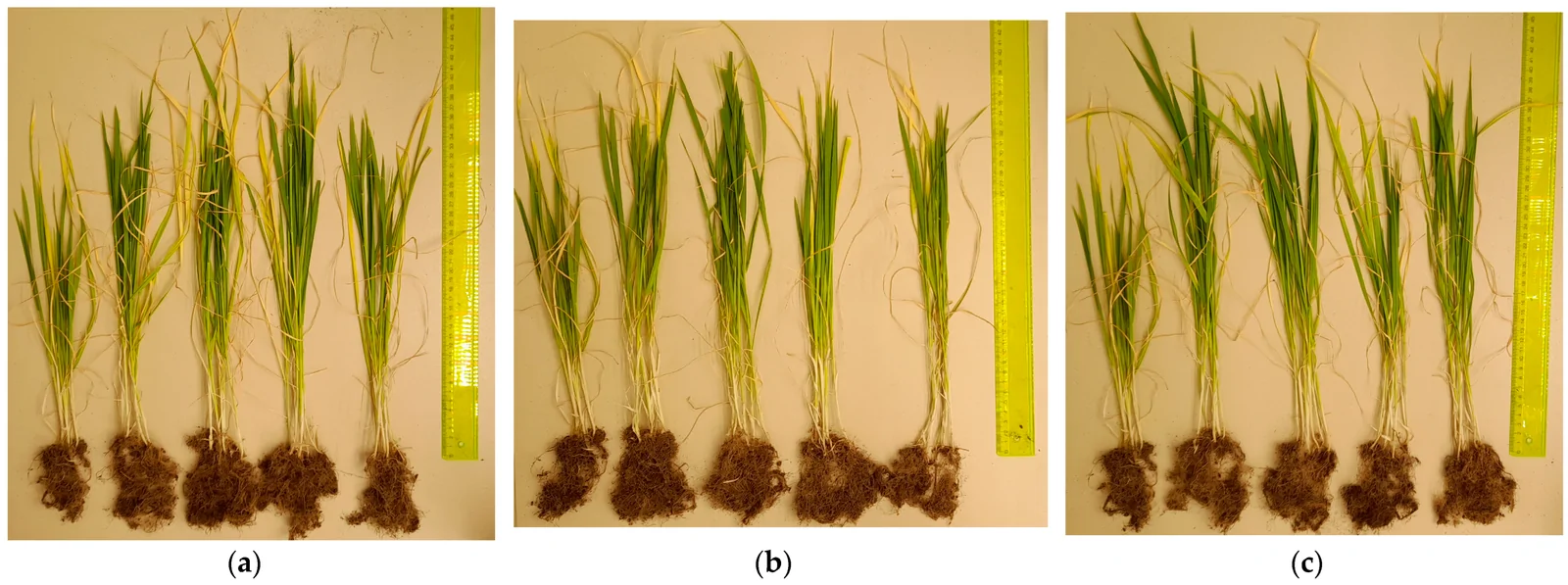
Wheat grown under model conditions in the presence of heavy metals, after completion of a 6-week growing experiment: (**a**) control, I, I + W14, I + W21, I + W28; (**b**) control, W0, W14, W21, W28; (**c**) control, W14 + 21, W14 + 28, W21 + 28, W14 + 21 + 28.

**Figure 3:**
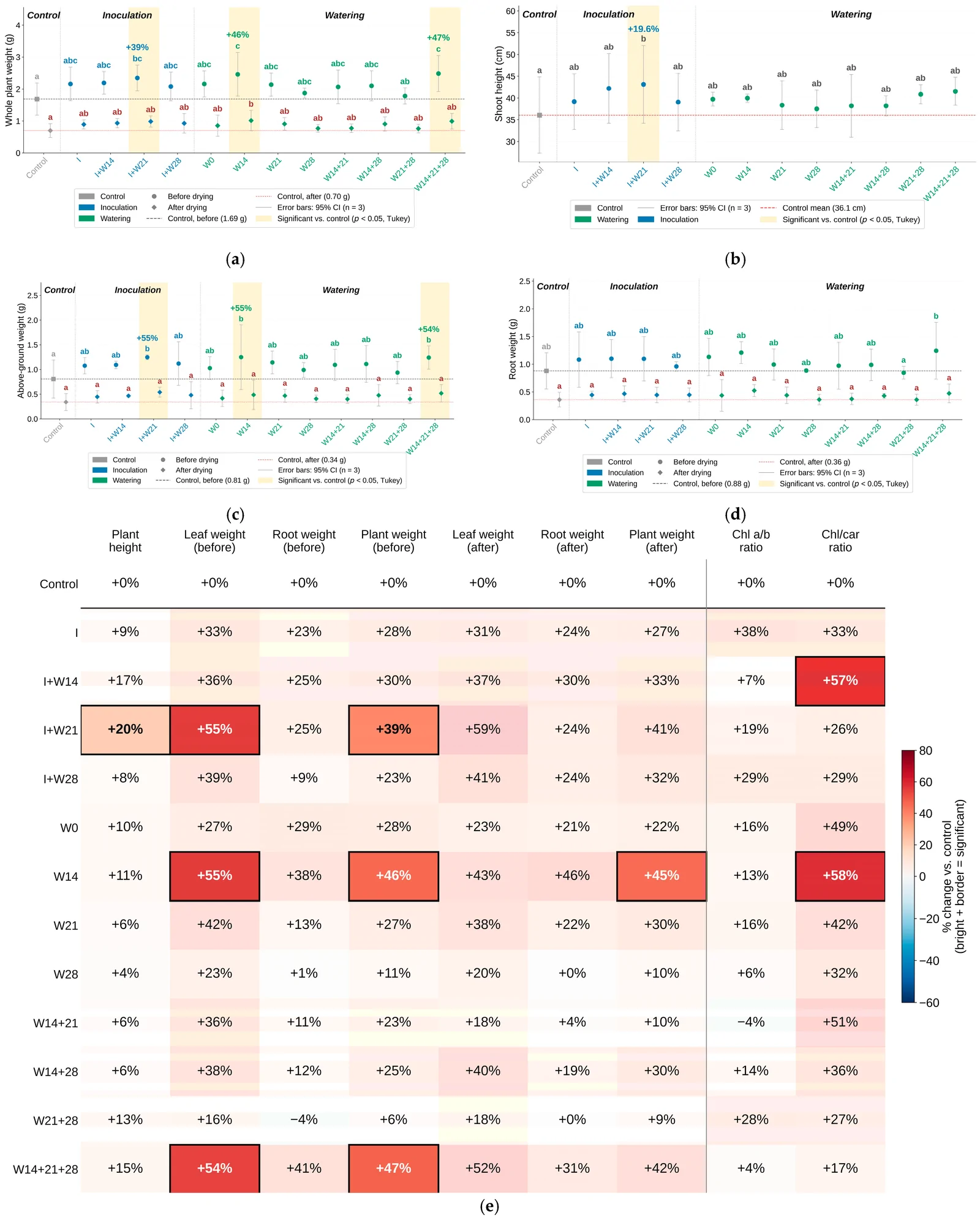
Growth parameters of wheat at the end of the 6-week pot experiment under heavy metal stress: (**a**) mass of a single plant; (**b**) shoot height; (**c**) mass of the above-ground part of a single plant; (**d**) root mass of a single plant; (**e**) heat map showing changes in characteristics as a percentage of the control. Intense shading indicates statistically significant deviations from the control; pale background indicates no statistically significant difference. The increase relative to the control is shown as a percentage. Application variants are grouped by method (Control, Inoculation, Watering; italic labels, separated by dashed vertical lines). Different lowercase letters above each point indicate statistically significant differences between application variants within the same panel (Tukey HSD, *p* < 0.05); variants sharing at least one letter do not differ significantly. In (**e**), cell shading intensity and a bold border indicate a statistically significant difference from the control (Tukey HSD, *p* < 0.05); pale-shaded cells are not significant.

Based on the results of a six-week growing experiment conducted under conditions with elevated levels of heavy metals in the soil, three treatment types had a significant positive effect on wheat. These treatments included inoculation with additional watering on day 21, a single watering on day 14, and three waterings on days 14, 21, and 28 (see Figure 2 and Figure 3).

With inoculation followed by additional watering on day 21 after planting, an increase in height was observed by 19.6% (43.13 ± 3.60 cm versus 36.07 ± 3.52 cm), and the total fresh weight of the plant by 39% (2.35 ± 0.14 g versus 1.69 ± 0.29 g). At the same time, the increase in the plant’s mass was due solely to an increase in above-ground mass of 54% (1.25 ± 0.02 g versus 0.81 ± 0.16 g).

With a single watering on the 14th day after planting, the total weight of the plant increased by 46% (2.46 ± 0.33 g versus 1.69 ± 0.29 g). At the same time, the increase in the plant’s mass was also due solely to an increase in above-ground mass of 54% (1.25 ± 0.26 g versus 0.81 ± 0.16 g).

With repeated application of the product (on days 14, 21, and 28 after planting), an increase in plant weight by 47% (2.49 ± 0.30 g) was observed, which was due to growth of both the above-ground part, by 53% (1.24 ± 0.09 g), and the root part, by 41% (1.24 ± 0.21 g).

Overall, this suggests that in the presence of heavy metals, the growth of the above-ground part of wheat requires watering with a suspension of *Bacillus subtilis* GMG1288 during periods of increased stress exposure, such as days 14 and 21. The greatest overall increase in both above-ground and root mass was achieved with multiple waterings on days 14, 21, and 28.

#### 2.3.2. Soil Salinity (NaCl)

**Figure 4:**
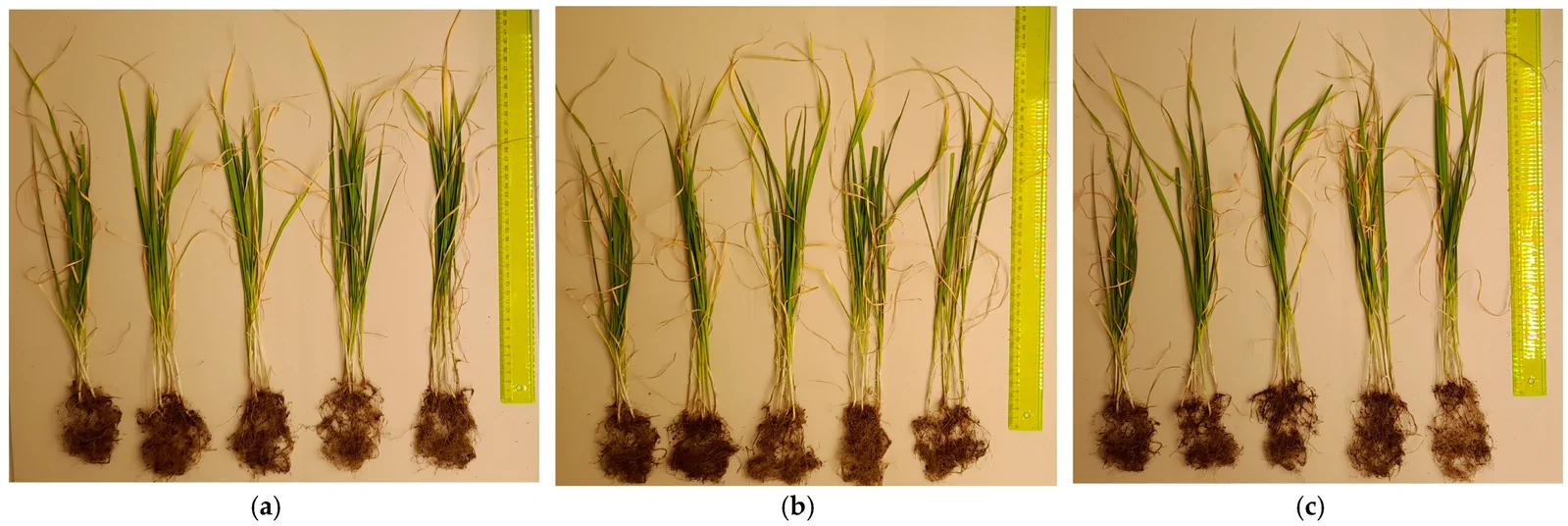
Wheat grown under simulated soil salinization conditions after a 6-week growing experiment: (**a**) control, I, I + W14, I + W21, I + W28; (**b**) control, W0, W14, W21, W28; (**c**) control, W14 + 21, W14 + 28, W21 + 28, W14 + 21 + 28.

**Figure 5:**
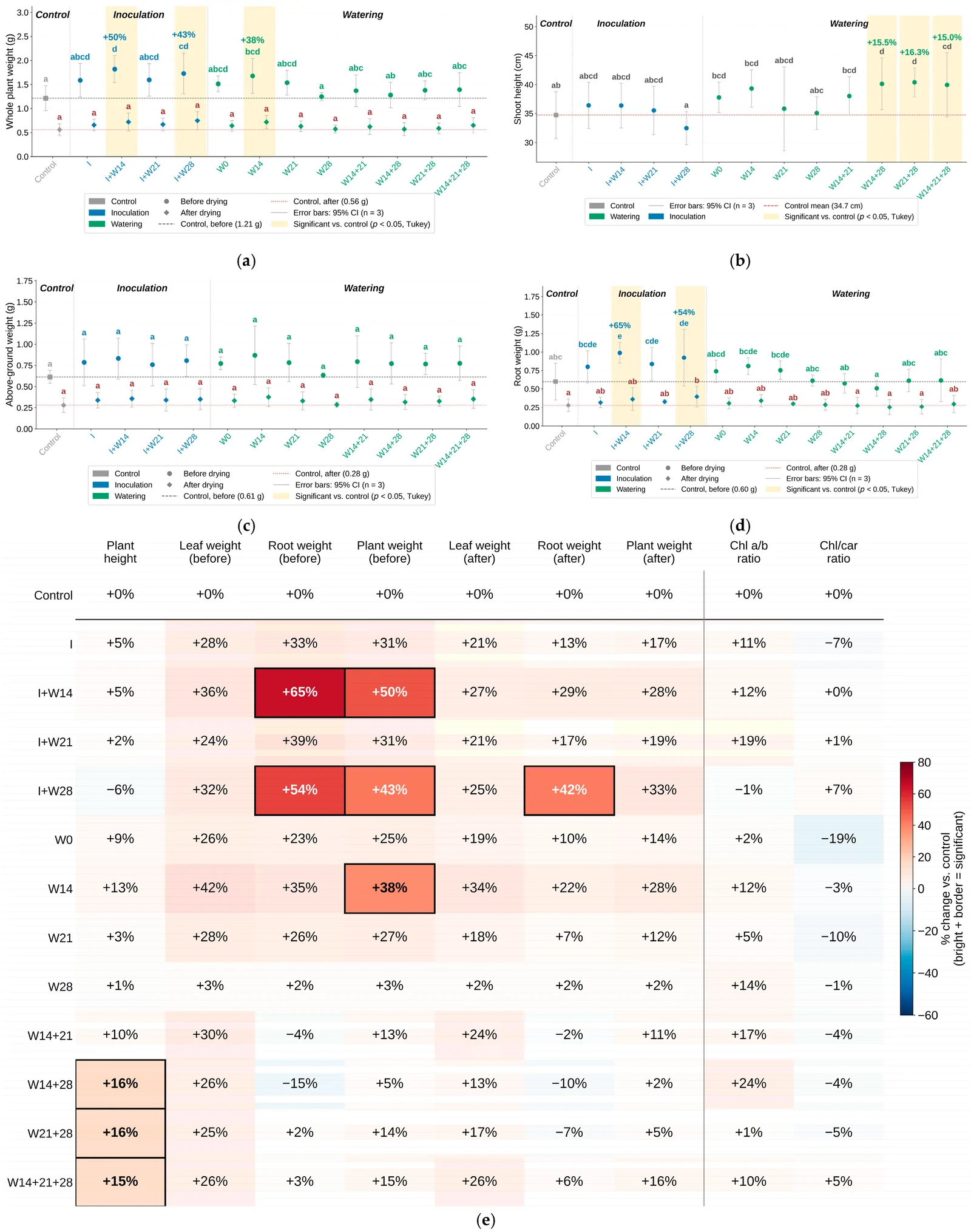
Growth parameters of wheat at the end of the 6-week pot experiment under soil salinity stress: (**a**) mass of a single plant; (**b**) shoot height; (**c**) mass of the above-ground part of a single plant; (**d**) root mass of a single plant; (**e**) heat map of parameter changes as a percentage of the control. Intense shading indicates statistically significant deviations from the control; pale background indicates no statistically significant difference. The increase relative to the control is shown as a percentage. Application variants are grouped by method (Control, Inoculation, Watering; italic labels, separated by dashed vertical lines). Different lowercase letters above each point indicate statistically significant differences between application variants within the same panel (Tukey HSD, *p* < 0.05); variants sharing at least one letter do not differ significantly. In (**e**), cell shading intensity and a bold border indicate a statistically significant difference from the control (Tukey HSD, *p* < 0.05); pale-shaded cells are not significant.

Under soil salinity conditions, a significant increase in root mass relative to the stress control was observed only with seed inoculation and additional watering on day 14 (I + W14: +65%, 0.99 ± 0.06 g versus 0.60 ± 0.10 g; *p* < 0.05) and with inoculation and additional watering on day 28 (I + W28: +53%, 0.92 ± 0.15 g; *p* < 0.05). Single seed inoculation (+33%, 0.80 ± 0.09 g) and inoculation with watering on day 21 (+40%, 0.84 ± 0.09 g) showed a comparable increase in root mass, but did not reach statistical significance (Figure 4 and Figure 5).

Watering wheat with the bacterial suspension without inoculation under soil salinity conditions led to an increase in shoot height; however, despite statistical significance, the percentage increase was not substantial (ranging from 15.0% to 16.3%). The increase in plant mass occurred primarily through an increase in root mass and was observed only with suspension watering on days 14 and 21 (root mass 0.81 ± 0.05 g, a 35% increase, and 0.75 ± 0.05 g, a 25% increase, respectively).

In summary, under soil salinity conditions, inoculation of wheat seeds with a *Bacillus subtilis* GMG1288 suspension increased root mass, and additional watering enhanced this effect. Watering without inoculation affected above-ground mass gain, and only watering immediately before stress onset (on day 14) increased root mass; however, the magnitude of this effect was smaller than that observed with inoculation.

#### 2.3.3. Osmotic Stress (PEG, Drought Model)

**Figure 6:**
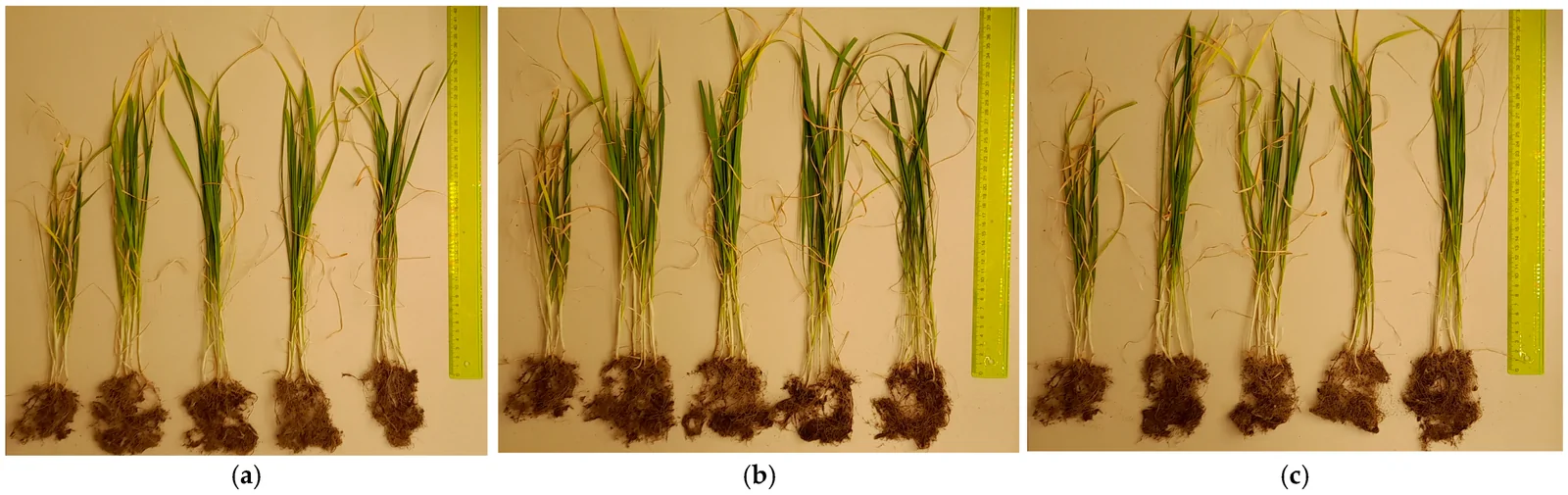
Wheat grown under osmotic stress after a 6-week growing experiment: (**a**) control, I, I + W14, I + W21, I + W28; (**b**) control, W0, W14, W21, W28; (**c**) control, W14 + 21, W14 + 28, W21 + 28, W14 + 21 + 28.

**Figure 7:**
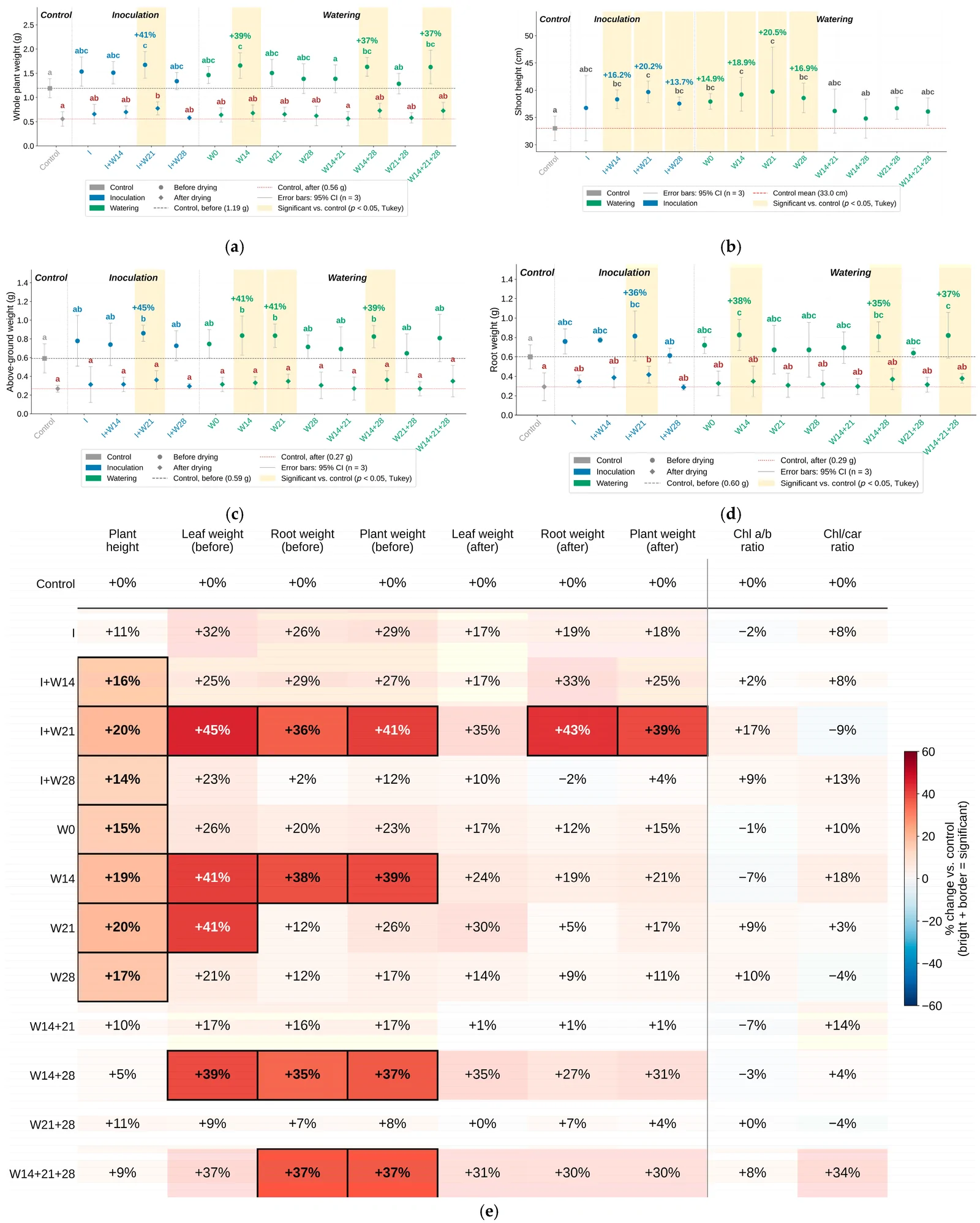
Growth parameters of wheat at the end of the 6-week pot experiment under osmotic stress: (**a**) mass of a single plant; (**b**) shoot height; (**c**) mass of the above-ground part of a single plant; (**d**) root mass of a single plant; (**e**) heat map of parameter changes as a percentage of the control. Intense shading indicates statistically significant deviations from the control; pale background indicates no statistically significant difference. The increase relative to the control is shown as a percentage. Application variants are grouped by method (Control, Inoculation, Watering; italic labels, separated by dashed vertical lines). Different lowercase letters above each point indicate statistically significant differences between application variants within the same panel (Tukey HSD, *p* < 0.05); variants sharing at least one letter do not differ significantly. In (**e**), cell shading intensity and a bold border indicate a statistically significant difference from the control (Tukey HSD, *p* < 0.05); pale-shaded cells are not significant.

Under PEG-induced osmotic stress, treatment with *Bacillus subtilis* GMG1288 during the period of increased stress activity (days 14 and 21) had the greatest effect on wheat (Figure 6 and Figure 7). Under these conditions, the increase in plant mass was achieved through a combined increase in both above-ground and root mass. With inoculation and additional watering after the onset of stress (on day 21), plant mass was 1.68 ± 0.14 g (a 43% increase, versus 1.19 ± 0.11 g), above-ground mass was 0.86 ± 0.03 g (a 46% increase, versus 0.59 ± 0.06 g), and root mass was 0.82 ± 0.10 g (a 37% increase, versus 0.60 ± 0.05 g). With watering immediately before stress onset (on day 14), plant mass was 1.66 ± 0.11 g (a 39% increase), above-ground mass was 0.84 ± 0.08 g (a 42% increase), and root mass was 0.83 ± 0.06 g (a 38% increase).

Additional watering with the suspension at later time points did not provide any further benefit. With watering on days 14 and 28, plant mass was 1.63 ± 0.10 g (a 37% increase), above-ground mass was 0.83 ± 0.05 g (a 41% increase), and root mass was 0.81 ± 0.06 g (a 35% increase). With watering on days 14, 21, and 28, plant mass was 1.63 ± 0.19 g (a 37% increase) and root mass was 0.82 ± 0.09 g (a 37% increase).

A single watering with *Bacillus subtilis* GMG1288 under osmotic stress conditions increased the height of the above-ground plant part from 13.7% to 20.5%, reaching its maximum value with watering on day 21. The inoculation and watering on day 21 variant reached 39.68 ± 0.79 cm (a 20.2% increase, versus 33.00 ± 0.90 cm), and the watering-only on day 21 variant reached 39.75 ± 3.28 cm (a 20.5% increase).

In summary, under osmotic stress conditions, inoculation with watering 3 days after the peak stress load (on day 21) had the best balanced effect on plant mass, while additional late waterings did not further improve growth parameters. Single waterings at any time point increased shoot height, with the best results observed for watering on day 21.

#### 2.3.4. Approach to Unstressed Control Levels

Since the key objective of treatment with the biological product *Bacillus subtilis* GMG1288 is to reduce the negative impact of abiotic stress on wheat, it is important to evaluate not only the increase relative to the stress control (plants subjected to the same stress but without treatment), but also to compare the parameters of treated plants with the unstressed control—plants grown without stress and without treatment. Such a comparison makes it possible to assess the direction of the effect: whether, under the influence of treatment, the plant approaches its normal, unstressed parameters.

**Figure 8:**
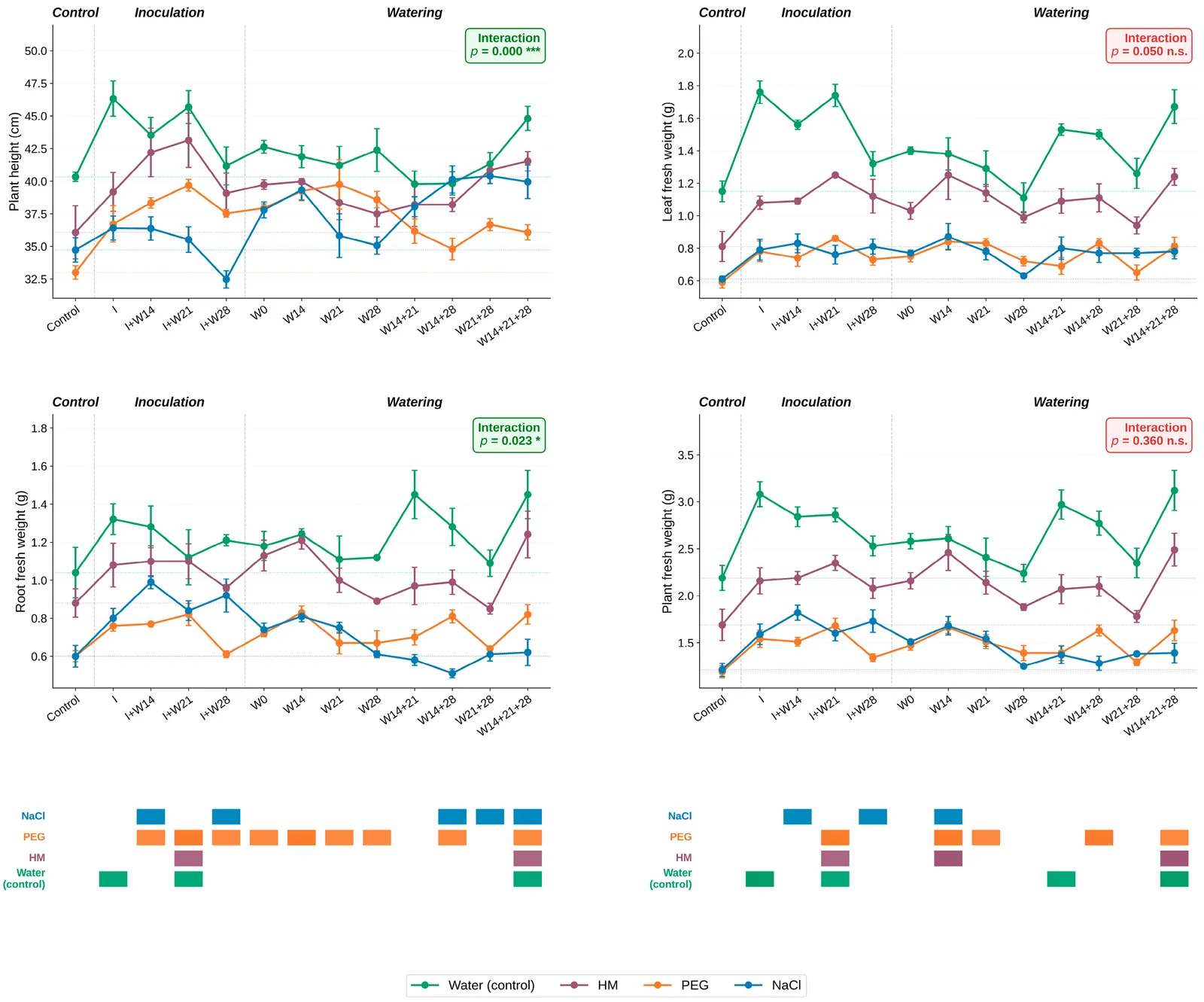
Summary graphs showing the effect of various types of wheat treatment with the *Bacillus subtilis* GMG1288 strain on wheat height and weight. The colored rectangles under the graph indicate the achievement of statistical significance (*p* < 0.05, Tukey HSD) compared to the corresponding stress control, color-coded by stress condition as in the legend. The boxed value in each panel shows the *p*-value for the stress × application method interaction term (two-way ANOVA); *** *p* < 0.001, * *p* < 0.05, n.s. = not significant (*p* ≥ 0.05).

Figure 8 shows that all the statistically significant treatment effects described above shift wheat parameters toward the unstressed control. However, this comparison is descriptive in nature: a formal statistical equivalence test (e.g., TOST with predefined bounds) between stress-treated plants and the unstressed control was not performed, as the small sample size of the unstressed control (n = 3) would give statistical power too low to confirm equivalence.

#### 2.3.5. Overall Assessment of the Application Method Independent of Stress Type

Given that many treatment variants were composite in nature, the possibility cannot be excluded that the observed effect was driven solely by the presence of a particular application type. The categorical analysis of variance performed (Table 2) directly supports this hypothesis: the application method (inoculation with or without watering/single watering/multiple watering) had a statistically significant effect on leaf, root, and total plant mass, with the effect depending on the type of stress (a significant method × stress interaction, *p* = 0.001–0.007).

The decomposition of application-timing effects showed that inoculation was the key contributor to root development under salt stress, whereas under osmotic stress and heavy metal contamination, the greatest effect on both root and above-ground mass was achieved by applying the product immediately before the onset of stress, on day 14. The only application method effective solely as a combination was triple watering on days 14, 21, and 28 under heavy metal contamination: only repeated watering led to consistent growth across all wheat parameters.

#### 2.3.6. Overall Assessment of Application Timing Across Stress Types

**Figure 9:**
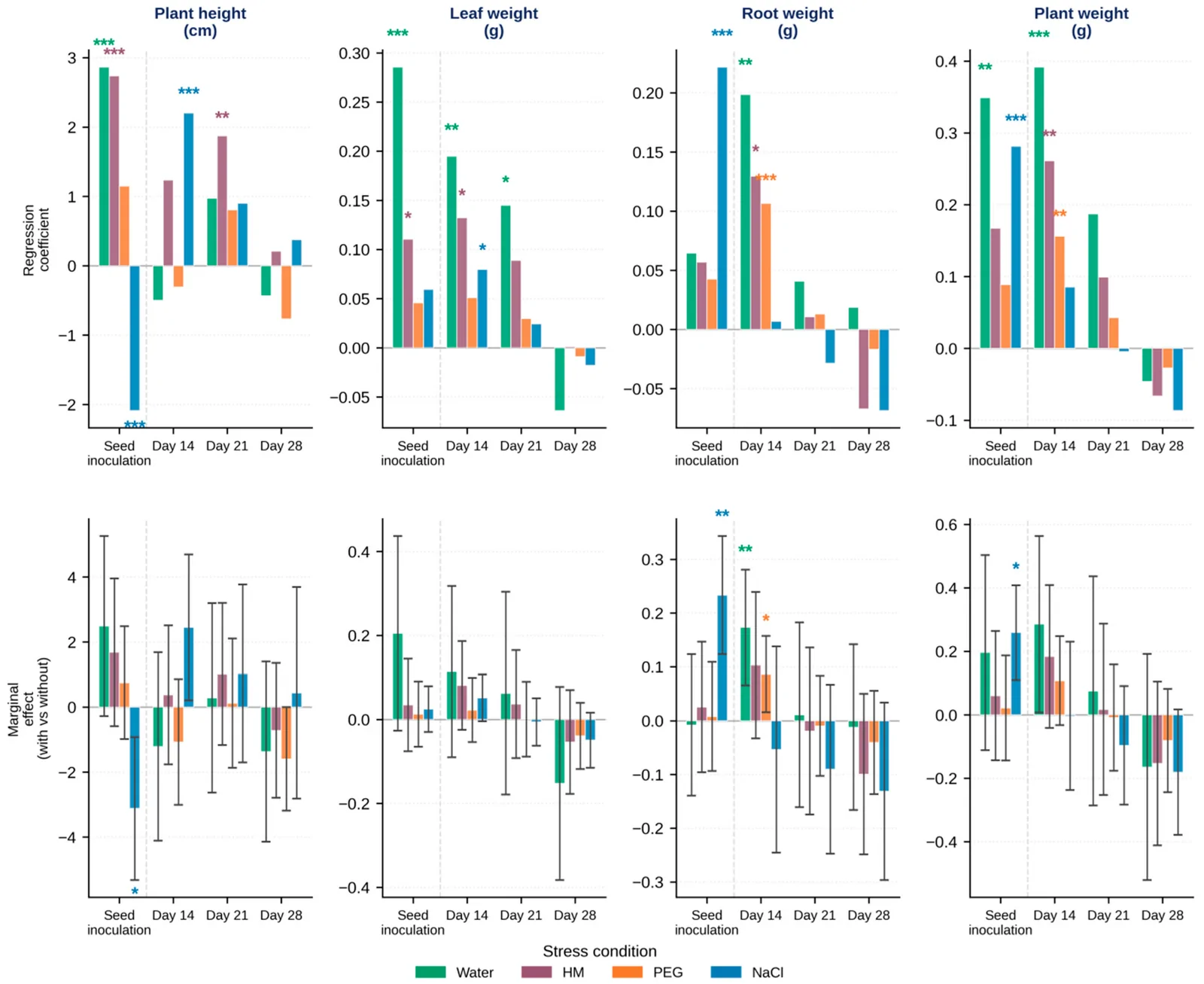
Decomposition of the effect of the application timing. The four upper graphs show the regression coefficient β for each predictor, which reflects the independent contribution of the corresponding application timing to the measured indicator, with fixed values of the other predictors. The four lower graphs show the difference in mean indicator values between the group of variants containing this timing and the group of variants not containing it. Statistical significance is indicated by the number of asterisks: * *p* < 0.05, ** *p* < 0.01, *** *p* < 0.001; bars without an asterisk are not statistically significant (*p* ≥ 0.05).

Regression decomposition (Figure 9) estimates the independent contribution of each application timing separately for each stress type. To test whether a general pattern exists that is independent of stress type, we additionally performed a three-way analysis of variance (application timing × presence of inoculation × stress) on a subsample of single-application variants (day 0/14/21/28, with or without inoculation). The “timing × stress” and “timing × inoculation” interactions were not significant (*p* > 0.06 in all cases, Table 2)—that is, the effect of application timing was consistent in direction regardless of stress type and regardless of whether the application was accompanied by seed inoculation.

**Figure 10:**
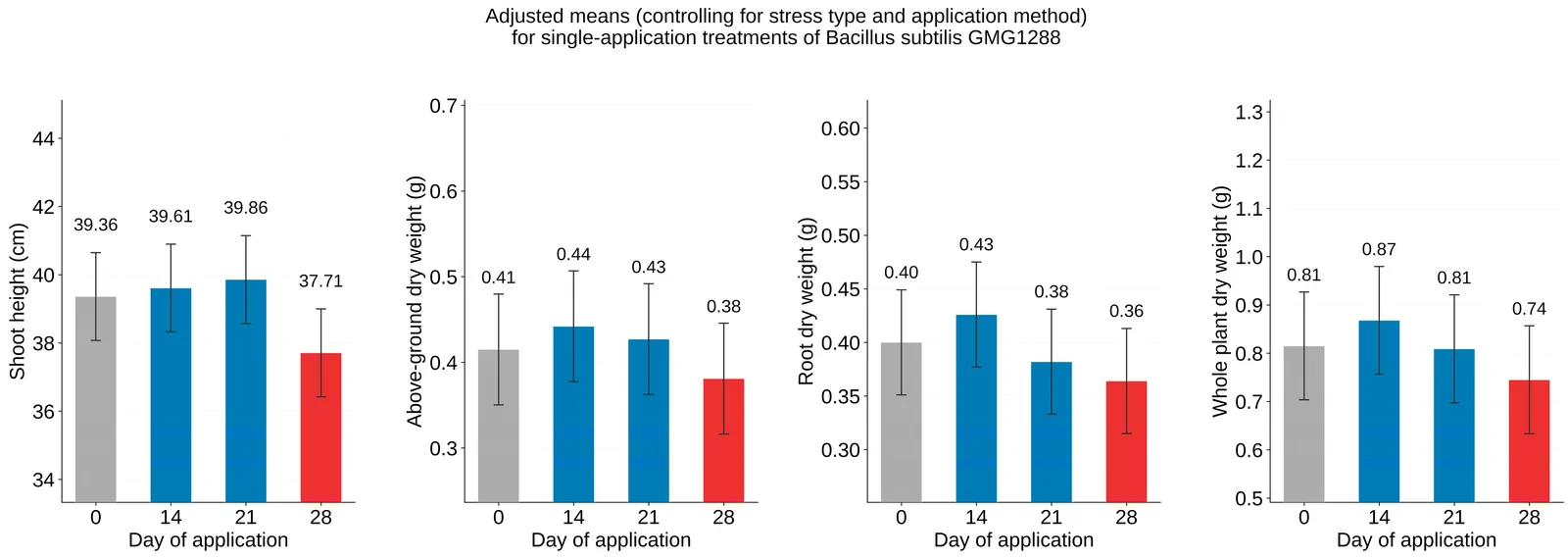
Adjusted means (controlling for stress type and application method) for single-application treatments of *Bacillus subtilis* GMG1288. Bar color indicates Tukey HSD grouping: gray = day 0, blue = days 14 and 21 (not significantly different from each other), red = day 28 (significantly lower than days 14 and 21; Tukey HSD, *p* < 0.05). Error bars show 95% confidence intervals.

After statistical adjustment for the effects of stress and inoculation, post hoc comparison (Tukey HSD) showed that application on day 28 resulted in significantly lower values than application on days 14 and 21 in terms of shoot height, root mass, and total plant mass (*p* = 0.004–0.017), whereas days 14 and 21 were not statistically distinguishable from each other and both consistently outperformed day 28 (Figure 10). Day 0 (inoculation without watering/watering on the day of planting) occupied an intermediate position, showing no statistically significant difference from either the near-stress-onset window (days 14–21) or the “late” application window (day 28).

Thus, within the range of stress conditions modeled in this experiment and regardless of the application method, the window of application at the onset of stress (days 14–21 after planting) represents the optimal timing for *Bacillus subtilis* GMG1288 treatment, whereas delayed application (on day 28) systematically produces the weakest effect.

## 3. Discussion

The effect of the *Bacillus subtilis* GMG1288 strain on the growth of spring bread wheat (*Triticum aestivum* L.) under different application regimes and abiotic stress conditions is characterized by a clearly pronounced differentiation of effects depending on the method and frequency of treatment, as well as on the type of stress agent. The results for the three stresses studied differed substantially—both in which plant parameters responded to treatment (height, root mass, above-ground mass) and in the degree to which the effect depended on the application method and timing (Section 2.2). Such context-dependent efficacy of PGPB is a common phenomenon: the success of a bioinoculant is determined by a complex combination of strain properties, host plant status, and environmental conditions, rather than by a single universal mechanism [11]. In particular, the timing of inoculant application relative to other events—colonization of the rhizosphere by competitors, the onset of stress exposure—may determine whether the introduced strain manages to establish itself and exert its effect; this phenomenon, known as the priority effect, has been described in detail both for microbial communities in general and for PGPM inoculants in particular [12].

It should be noted that all growth parameters measured in this study are exclusively phenotypic (height, fresh and dry biomass of shoots and roots) and do not capture the physiological and biochemical changes occurring in plants under abiotic stress—such as enzyme activity, antioxidant defense, or hormonal profiles. This was a deliberate scope limitation: phenotypic growth parameters directly determine the agronomic value of an application regimen, which is the primary practical objective of this work. The in vitro phenotypic characterization of the strain (Section 2.1) was likewise obtained without replication and is descriptive in nature; it reflects the strain’s tolerance profile and selected PGPB traits but does not constitute a complete mechanistic characterization. In particular, ACC-deaminase activity, exopolysaccharide production, and ion homeostasis modulation were not assessed, which limits mechanistic inference under stress conditions.

### 3.1. Mechanisms of Action of Bacillus subtilis GMG1288 by Stress Type in Triticum aestivum L.

#### 3.1.1. Salt Stress—Root Colonization

In our study, application of *Bacillus subtilis* GMG1288 induced an increase in wheat root system mass specifically under salt stress, with the effect being localized—without a corresponding change in shoot height—which indicates action in the zone of direct contact between the bacterium and the root, rather than a systemic effect. It should be noted, however, that this interpretation is correlational rather than causal: the physiological measurements that would have allowed the contribution of root colonization to be distinguished from other candidate mechanisms—such as osmoprotection or phosphate mobilization—were not performed in this study.

One aspect worth highlighting is that part of the observed increase in root mass under salt stress pertains to fresh rather than structural (dry) mass. The dry matter content of the root (the ratio of dry to fresh mass) in plants treated with *Bacillus subtilis* GMG1288 under NaCl stress was systematically lower than in the untreated stress control (≈0.37–0.43 versus 0.467)—that is, the tissue of treated roots contains relatively more water. This may reflect a more favorable tissue water status (which is consistent with the strain’s high proline production in vitro, since proline is involved in osmoregulation and water retention); however, without direct measurement of tissue water status and root architecture, this remains a hypothesis rather than an established mechanism.

The most pronounced root system response was observed with seed inoculation, which supports root colonization by the bacterium as the most plausible explanation for the localized effect. This mechanism has been documented in detail for related *B. subtilis* strains in several plant species, including wheat under salinity conditions, where the increase in root length grew proportionally with the intensity of salt stress—which is qualitatively consistent with the results of the present study [7,13].

The key difference between inoculation and watering lies not so much in the chemical composition of the medium as in the mode of bacterial delivery to the root. With watering, cells are introduced into the bulk of the substrate and must independently traverse a certain distance to reach the root surface in order to colonize it; with seed inoculation, the bacterium is brought into direct contact with the developing root system already at the moment of germination, bypassing the stage of directed movement through the substrate. The ability of a cell to efficiently traverse such a distance may depend on the production of biosurfactants (in particular, surfactin), which enable swarming-type motility across surfaces and through the soil pore space: it has been shown that *B. subtilis* strains defective in the sfp gene lose the ability to spread across surfaces and form structured multicellular communities [14]. This observation is critically important in light of the selection of agricultural strains: the strain must retain the ability to secrete surfactins under salinity conditions in order to improve root penetration during watering.

At the same time, Steil et al. demonstrated repression of *B. subtilis* chemotaxis and motility genes (in particular, the flagellar protein gene hag—by 21-fold) at 1.2 M NaCl (≈7%) [8]. This may explain the consistent advantage of inoculation for root mass specifically under salt stress, if salinity additionally impedes the bacterium’s ability to independently reach the root.

The soil substrate used in this experiment was not sterilized. This was a deliberate choice reflecting the applied objective of the study: to evaluate application regimen efficacy under conditions approximating real agronomic practice, where soil sterilization is neither performed nor technically feasible at field scale. The observed effects therefore reflect the outcome of GMG1288 application against the background of a native soil microbial community. This design does not allow direct effects of the introduced strain to be distinguished from indirect effects mediated through interactions with the resident microbiota—competition, synergism, or shifts in community composition. Soil pH and other physicochemical soil parameters were not measured; accordingly, the potential influence of stress agents on soil properties cannot be assessed within the framework of this experiment. Both aspects are acknowledged as limitations of the present study.

#### 3.1.2. Heavy Metals—From Failed Colonization to Cell Survival

Unlike wheat seed inoculation with *Bacillus subtilis* GMG1288, which promoted root growth under salt stress, the same treatment did not produce a comparable effect under soil contamination with heavy metals.

This phenomenon is partly explained by the study of Falcón García C et al., who showed that Cu^2+^ and Zn^2+^ ions do not substantially affect planktonic growth of *B. subtilis*, but fundamentally alter the structure of the biofilms formed: in the presence of CuSO_4_, expression of the matrix genes *tasA* and *bslA* is markedly reduced, whereas in the presence of ZnCl_2_, tapA is predominantly suppressed. The biofilm formed in the presence of heavy metals loses its hydrophobic properties, and the bacterial culture becomes susceptible to antibiotics. Thus, heavy metals disrupt precisely those components of the extracellular matrix that are critical for root colonization, leaving overall bacterial growth intact while depriving the cells of their ability to form a stable biofilm on the root surface [9]. Data on the intrinsic growth of GMG1288 partially agree with this picture: in the presence of Cu, strain growth did not differ from the control (121.93 ± 20.20%, Section 2.1), which is consistent with Arnaouteli et al.’s claim regarding the intactness of planktonic *B. subtilis* growth under Cu^2+^ exposure. However, in the presence of Zn, GMG1288 growth was substantially suppressed (15.29 ± 9.96%, Section 2.1)—this discrepancy may point to a strain-specific increased sensitivity of GMG1288 to zinc. Thus, the absence of a pronounced effect on wheat root mass under heavy metal stress in the pot experiment (Section 2.3.1) can be attributed both to disruption of the biofilm matrix (Cu) and to direct inhibition of bacterial growth (Zn).

This is also consistent with the application scheme that showed the greatest effectiveness: multiple watering with the suspension—the only scheme that produced a stable effect under soil contamination with heavy metals—represents a fundamentally different mode of product application compared to single seed inoculation. This further supports the notion that the mechanism underlying the effect under this type of stress is most likely different from root colonization.

The literature describes several mechanisms by which PGPB can mitigate heavy metal toxicity to plants [15]. From the standpoint of their effect on the plant, these can be reduced to two fundamentally different pathways. The first is a reduction in metal bioavailability in the soil through biosorption, precipitation, or redox transformation of the metals themselves. For example, *B. subtilis* BM2, despite morphological cell damage caused by Ni and Pb—verified by scanning and transmission electron microscopy, as well as confocal laser scanning microscopy—retained viability and demonstrated the ability to accumulate metals within intracellular structures [16]. A key mechanism ensuring bacterial survival under metal stress was the induction of metallothionein synthesis—low-molecular-weight, cysteine-rich proteins whose thiol groups bind heavy metal ions, thereby reducing their bioavailability. It is possible that repeated applications of bacterial biomass allow a greater quantity of metal ions to be sorbed onto bacterial cells, thereby alleviating stress for the plant.

The second pathway is the ability of the bacterial cell to survive in the presence of the metal (through intracellular detoxification mechanisms—efflux systems, metallothioneins, and others) and, remaining viable, to continue exerting a growth-promoting effect on the plant—for example, through phosphate mobilization or phytohormone production [17]. If this is the case, then with repeated application, the total number of viable cells in the rhizosphere may be maintained at a sufficient level, and even in the free (planktonic) state, without direct contact with the root, these cells may exert a growth-promoting effect, for example, through phosphate-mobilizing activity.

#### 3.1.3. Osmotic Stress—Hormonal Blockade at the Plant Level

Strain GMG1288 shows practically undetectable IAA production (Section 2.1). Consequently, the IAA-mediated hormonal stimulation of root growth—a key mechanism for many other *B. subtilis* strains [10]—does not operate in the present case. The inclusion of osmotic stress in the experimental design was nevertheless deliberate: the objective was to assess whether other PGPB activity mechanisms of GMG1288—in particular, osmoprotection via proline production and phosphate mobilization—are operative under this stress type, and whether application method and timing modulate their expression.

The observed temporal dynamics of treatment efficacy under osmotic stress—the greatest effect when applied immediately before stress onset or right upon reaching full intensity (days 14 and 21), and the smallest effect after eight days of continuous exposure to full-intensity stress (day 28; Section 2.3.5)—suggests that what matters is not so much the state of the bacterium itself at the time of application (the intensity of stress in the medium no longer differs between days 21 and 28), but rather the state of the host plant at that time. A plausible explanation here may be the following mechanism—hormonal reprogramming of the plant under osmotic stress: under PEG conditions, wheat develops a hormonal imbalance, with ABA accumulation accompanied by a decline in IAA and cytokinins. Against this background, additional bacterial application apparently no longer has an effect: if the plant’s hormonal balance has by this point shifted toward ABA dominance, the plant likely loses the capacity to respond to bacterial growth-promoting signals, regardless of how much fresh suspension is applied [10]. Since GMG1288 does not produce IAA, the strain cannot itself compensate for the auxin deficit that develops under ABA dominance—which is consistent with the generally weak and timing-dependent effect observed under osmotic stress. Hormonal profiling of the plants was not performed in the present study, so this mechanism is considered a plausible but independently unconfirmed contribution.

It should also be noted that osmotic stress in this study was modeled using a PEG solution rather than progressive soil drying. The identified window of maximum efficacy (days 14–21) is valid specifically for this experimental model and requires separate verification before being generalized to drought in the broader agronomic sense: PEG-induced and soil-drying-induced osmotic stresses differ in the spatial distribution of water potential gradients in the root zone, and the optimal timing of PGPB application may differ accordingly.

### 3.2. Application Method and Timing

A categorical analysis (Section 2.2, Table 2) showed that both application method and timing significantly influence the effect—but not equally across different stresses and, more importantly, not equally relative to each other. Comparing the two factors across the three stresses reveals not a single “primary lever,” but a stress-specific one. It should be noted that all statistical conclusions in this section are based on a minimal sample size (n = 3 biological replicates per variant), which limits the power to detect interactions between factors; the corresponding results should therefore be interpreted as indicative of directional patterns rather than as definitive quantitative estimates. A similar stress- and method-specific pattern has also been noted for other PGPB systems: a comparison of application methods for the same marine PGPB consortium in tomato showed that single seed inoculation conferred the lowest heat stress tolerance compared to foliar spraying and encapsulated delivery [18]—that is, the preferability of a particular application method is itself dependent on the specific “strain–stress” system, rather than being a general property of one universally superior method. Similarly, the same Pseudomonas sp. strain exhibited efficacy that differed both in magnitude and in which plant traits were affected in rice under drought versus salinity, even though both stresses produce qualitatively similar secondary effects [19]—which underscores the need to tailor the application regime to the specific stress rather than transferring it by analogy.

Under salt stress, the application method proves decisive: inoculation significantly outperforms multiple watering in terms of root and total plant mass, whereas differences between individual application timings are less pronounced—day 28 is not even the worst among the four timings here. This is consistent with the general logic of the priority effect in microbial inoculants [12] and with the practice of timing the application of *B. subtilis*-like PGPR to specific plant developmental stages under salt stress, since the timing of application relative to plant developmental stage is recognized as particularly critical for this type of stress [20].

Under osmotic stress, by contrast, timing proves decisive: the application method does not differ statistically for any of the mass parameters (it is significant only for height), whereas the influence of timing is the sharpest among all three stresses (the effect on day 28 is nearly nullified). This is consistent with the proposed mechanism, in which the bottleneck is not bacterial delivery by one method or another, but the hormonal state of the plant itself (ABA accumulation, decline in IAA and cytokinins [10]—if the plant’s “window of responsiveness” has closed, the application method of the bacterium no longer matters.

Under heavy metal stress, the pattern cannot be reduced to either of the two factors: neither the method nor the timing differs statistically when analyzed categorically (all pairwise comparisons by method are non-significant; the decline in effect by day 28 is the weakest among the three stresses). At the same time, individual variants—watering on day 14, inoculation with watering on day 21, and triple watering on days W14 + 21 + 28—showed a pronounced effect. This is consistent with independent data: introduced PGPB generally establish poorly in metal-contaminated soil and produce only minor changes in the composition of the soil microbial community [21]—that is, the effect is likely determined not by a single successful match of method or timing, but by the cumulative, continuously maintained number of viable cells in the rhizosphere.

### 3.3. Practical Implications

The development of an application protocol for a PGPB-based product cannot rely solely on trying out combinations of method and timing—without understanding the mechanism through which the strain exerts its effect under a specific stress, optimization risks remaining a chance finding that cannot be transferred to other growing conditions [11]. Notably, the very set of mechanisms available to GMG1288 rules out in advance some of the “standard” strategies that might have worked for other strains. For instance, this strain shows practically no detectable IAA production—meaning that strategies oriented toward hormonal stimulation of root growth by the bacterium itself (characteristic, for example, of auxin-active *Azospirillum* strains or certain *Pseudomonas*) cannot, in principle, serve as the main lever of efficacy for GMG1288, and attempts to optimize application timing specifically around this mechanism are bound to fail [22].

Instead, the leading properties for GMG1288 are phosphate mobilization and osmotolerance (proline)—and it is precisely these two traits, rather than a universal “PGPB profile,” that determine which application regime will work under a given stress. Likewise, the dependence of the effect on application method under salinity is meaningful only in light of the proposed colonization mechanism: if GMG1288 exerted its protective effect not through local contact with the root but, for example, through systemic induction of resistance at a distance, the application method (seed inoculation vs. watering) should not have mattered—yet it does, and this is precisely what supports the proposed mechanism, rather than merely describing an observation.

Thus, the observed patterns in application method and timing are not merely empirical optima, but consequences of a specific, though not fully established, mechanism of action; and it is precisely for this reason that PGPB application protocols should be developed not according to a single generic scheme of “inoculation vs. watering, early vs. late,” but based on a preliminary characterization of the mechanism of action of the specific strain for the specific stress—which, in turn, requires a minimal set of phenotypic data before field or greenhouse trials are conducted.

The stress-specific patterns identified here for application method and timing reflect the phenotypic profile of the specific strain GMG1288 and cannot be extrapolated to *Bacillus subtilis* as a species. Intraspecific variation in PGPB-relevant traits—IAA production, biofilm formation capacity, siderophore synthesis, ACC-deaminase activity—is well documented and substantial; results obtained for one strain therefore carry no predictive value for another. Application regimens for PGPB-based products should consequently be optimized individually for each strain, guided by its specific phenotypic activity profile rather than by generic method/timing schemes derived from other strains or species.

Several additional limitations of the present study should be noted in this context. The entire experiment was conducted on a single wheat cultivar (Novosibirskaya 31); since the efficacy of PGPB under abiotic stress has been reported to depend on host plant genotype, the patterns identified here may not be universal across wheat in general. The experiment represents a single pot-based trial with measurements taken at one time point, without replication across seasons and without field validation; this limits the generalizability of the findings and leaves assumptions about the temporal dynamics of the introduced bacterial population between application and stress onset untestable with the available data. These limitations notwithstanding, the stress-specific and mechanism-informed framework developed here provides a structured basis for designing more comprehensive multi-strain, multi-cultivar, and field-scale studies.

## 4. Materials and Methods

### 4.1. Plant Material and Pot Experiment Conditions

Spring wheat (*Triticum aestivum* L.) cultivar Novosibirskaya 31 was used as the plant material for this study. The pot experiment was conducted under standard greenhouse conditions (18-h photoperiod, average temperature 24 °C, ambient laboratory humidity without additional humidity control) for a duration of 6 weeks, with all four conditions (unstressed control and three stress variants) run in parallel within a single experimental cycle, at the same time points and using the same equipment and measurement protocols. Plants were grown in 8 × 8 × 8 cm pots (five plants per pot) containing a peat–perlite substrate (3:1, *v*/*v*) that was not sterilized prior to use and watered daily with water for the first 14 days. To model abiotic stress, four conditions were applied starting from day 15.

1. Control plants were watered with water without the addition of stress-inducing agents.
2. Salt stress was induced by gradually increasing the NaCl concentration in the irrigation water: 0.5% for the first 3 days, followed by 0.7% for the next 3 days, after which plants were switched to a maintenance regimen—a 1% NaCl solution applied 5 days per week for 3 weeks.
3. Osmotic stress was modeled using polyethylene glycol (PEG-6000, Sigma-Aldrich, Darmstadt, Germany) following an analogous adaptation scheme: 2% for 3 days, 3% for 3 days, followed by a 5% solution applied 5 days per week until the end of the experiment.
4. Heavy metal (HM) stress was induced by a stepwise increase in the concentration of a metal salt mixture: 0.5× for the first 3 days, 1× for the next 3 days, followed by 2× for 2 weeks, and 3× during the final week of the experiment (5 days per week). The base solution (1×) consisted of the following composition: (CH_3_COO)_2_Pb—0.2 g/L, CdCl_2_—0.005 g/L, ZnSO_4_—1.0 g/L, CuSO_4_—0.3 g/L.

The study uses two distinct control concepts: the “stress control”—a plant subjected to the same stress treatment (HM/PEG/NaCl) as the experimental variants, but not treated with the biological product; and the “unstressed control” (baseline)—a plant not subjected to stress and not treated with the biological product, measured within the same experimental cycle.

### 4.2. Bacterial Preparation and Application Scheme

A suspension of *Bacillus subtilis* GMG1288 was used as the biological agent. The preparation was applied at a concentration of 10^8^ CFU/mL. The experimental design included 13 application variants, grouped into three categories: (1) seed inoculation before sowing (I), (2) seed inoculation combined with additional watering with the suspension on day 14, 21, or 28 after sowing (I + W14, I + W21, I + W28), and (3) single or multiple watering with the suspension at various time points—on day 0, 14, 21, or 28, and their combinations (W0, W14, W21, W28, W14 + 21, W14 + 28, W21 + 28, W14 + 21 + 28). Control plants (stress control and unstressed control) were not treated with the preparation. Each variant included 3 biological replicates.

### 4.3. Determination of Growth and Weight Parameters

At the end of the pot experiment (week 6), shoot height (cm) was measured for each plant. For biomass determination, plants were divided into fractions: leaves, roots, and the whole plant. For each fraction, fresh mass (g) was determined by weighing immediately after separation, and dry mass (g) was determined after drying to constant mass at 70 °C.

### 4.4. Determination of Pigment Composition

The content of photosynthetic pigments was determined spectrophotometrically in a 96% ethanol extract according to the method of Lichtenthaler [23]. Chlorophyll a, chlorophyll b, and carotenoid content (mg/g fresh mass) were calculated, along with the chlorophyll a/b and chlorophyll/carotenoid ratios, which were used as integrative physiological indices of plant status.

### 4.5. In Vitro Phenotypic Characterization of the Strain

The growth rate of *Bacillus subtilis* GMG1288 was assessed in liquid culture at increasing concentrations of polyethylene glycol (1, 5, 10, 15%), NaCl (1, 5, 10, 15%), and heavy metal ions Cu, Cd, and Zn (1000 mg/L), and was expressed as a percentage of growth under control conditions (without a stress agent). Production of IAA [24], gibberellins [25], ammonium [26], salicylic acid [27], proline [28] and siderophores [29], phosphate-mobilizing activity [30] were determined using standard colorimetric methods, with three replicates per condition.

### 4.6. Statistical Data Analysis

All data are presented as mean ± standard deviation (M ± SD) of three biological replicates. Statistical analysis was performed in Python 3 using the statsmodels (version 0.14), scipy (version 1.11), and scikit-learn (version 1.8, for principal component analysis) packages.

Evaluation of differences between treatment variants. To compare the 13 application variants within each stress condition, one-way analysis of variance (one-way ANOVA) was applied. When significant differences were established (*p* < 0.05), post hoc pairwise comparisons were performed using Tukey’s HSD test. Results are presented using a compact letter display: variants that do not share a common letter differ significantly from each other at *p* < 0.05. On the figures, statistically significant differences from the stress control are additionally highlighted visually.

To visualize the combined data across all stress conditions, interaction plots were constructed, showing the mean ± standard error of the mean (SEM) for each measured parameter across the 13 application variants under the three abiotic stress conditions and the unstressed control (watering with water). Horizontal dashed lines correspond to the level of the unstressed control. A significance bar below the *X*-axis indicates variants that differ significantly from the stress control within the corresponding condition (Tukey’s test, *p* < 0.05).

Categorical analysis of application method. The 13 application variants were grouped into three application method categories: inoculation (with or without additional watering; 4 variants), single watering without inoculation (4 variants, on days 0/14/21/28), and multiple watering without inoculation (4 combined-day variants). The unstressed and stress controls were not included in this analysis. For each measured parameter (height, fresh and dry mass of leaves, roots, and the whole plant), a two-way analysis of variance (application method × stress, n = 12 per cell) was applied, followed by post hoc comparison of application methods using Tukey’s test, performed separately for each stress condition.

Categorical analysis of application timing. To test for a generalized effect of application timing independent of stress type, 8 single-application variants (inoculation and/or watering on day 0, 14, 21, or 28) were selected, and a three-way analysis of variance (timing × inoculation [yes/no] × stress, n = 3 per cell) was applied. Since the “timing × stress” and “timing × inoculation” interactions were not statistically significant, post hoc comparison of application timings was performed on residuals adjusted for the main effects of stress and inoculation (Tukey’s test on adjusted values). Adjusted means for each application timing were obtained as predicted values from the linear model val ~ C(stress) + C(inoc) + C(day), averaged across all combinations of stress and inoculation, and are used for visualization in Figure 10.

Decomposition of application-timing effects. Because most experimental variants included combinations of several application timings (e.g., 14 + 21 + 28), conventional pairwise analysis does not allow the independent contribution of each day to be estimated. To address this, each variant was encoded as a vector of four binary variables: seed inoculation (day 0), day 14, day 21, and day 28—each taking a value of 1 if the corresponding timing was included in the variant, and 0 otherwise. Based on this encoding, a linear regression model of the following form was constructed for each parameter and each stress condition:

Y = β_0_ + β_1_·inoc + β_2_·day14 + β_3_·day21 + β_4_·day28 + ε

The regression coefficient β for each predictor reflects the independent contribution of the corresponding application timing to the measured parameter, holding the other predictors constant. The significance of the coefficients was assessed using the *t*-test at *p* < 0.05. Additionally, for ease of interpretation, the marginal effect of each application timing was calculated as the difference in mean parameter values between the group of variants containing that timing and the group not containing it; significance was assessed using an independent two-sample Student’s *t*-test (scipy.stats.ttest_ind), with 95% confidence intervals constructed.

Principal component analysis (PCA). To visualize the overall structure of variability, all 7 measured parameters (height, fresh and dry mass of leaves, roots, and the whole plant) were standardized (z-normalized) and averaged across the three biological replicates for each “application variant × stress” combination (n = 52). PCA was used exclusively for visualization; statistical conclusions regarding the significance of effects were based on the analysis of variance described above.

Assessment of approach to the unstressed control. The comparison of stress-treated plants with the unstressed control is descriptive in nature (visualizing the direction of the effect). A formal statistical equivalence test (TOST, two one-sided t-tests with predefined bounds) was not performed for the present version of the manuscript, owing to the small sample size of the unstressed control (n = 3), at which such a test lacks sufficient statistical power even with wide equivalence bounds (±20% of the mean value of the unstressed control).

## 5. Conclusions

The present study demonstrates that the efficacy of *Bacillus subtilis* GMG1288 application stress type emerges as the dominant source of variability in wheat growth parameters, whereas application method and timing are statistically significant but substantially weaker and, importantly, stress-dependent modifiers of the effect: the “application method × stress type” interaction is significant across all measured mass and height parameters.

A stress-by-stress breakdown shows that this stress-dependence does not manifest uniformly, but rather as a shift in the “primary factor” governing the effect. Under soil salinity, application method proves decisive: seed inoculation produces the greatest increase in root mass, while the exact timing is secondary. Under osmotic stress, by contrast, timing proves decisive: treatment is effective only within a narrow window of days 14–21, regardless of application method, and later application is practically ineffective. Under heavy metal contamination, neither method nor timing alone yields a statistically robust effect—the most stable outcome is achieved with repeated application, which may reflect not a precise choice of regimen, but rather a gradual decline in the viable bacterial population in the toxic environment and the need for its regular replenishment.

A practical implication of this work is that the application regimen for a PGPB-based product should not be selected empirically or by analogy with other strains, but rather based on a pre-characterized activity profile of the specific strain. Accordingly, a minimal phenotypic characterization of the strain (in vitro resistance to the target stresses, production of key metabolites) should precede not only strain selection itself, but also the development of its application protocol—application method and timing are strain-specific characteristics, just as much as the composition of its growth-promoting activity, and cannot be transferred from one product to another without accounting for differences in the underlying mechanism.

## Figures and Tables

**Table 1:** Resistance to abiotic stresses in vitro and production of functional metabolites by the *Bacillus subtilis* GMG1288 strain (growth rate as a percentage of the control without a stress agent).

Stress	Growth Indicators, %			
Drought	1% PEG	5% PEG	10% PEG	15% PEG
	74.65 ± 35.0	40.04 ± 20.49	97.59 ± 67.72	43.66 ± 6.83
Salinity	1% NaCl	5% NaCl	10% NaCl	15% NaCl
	116.90 ± 11.95	50.30 ± 7.27	53.32 ± 8.52	0.44 ± 0.00
Heavy metals	Cu 1000 mg/L (15.74 mM)	Cd 1000 mg/L (8.90 mM)	Zn 1000 mg/L (15.29 mM)	
	121.93 ± 20.20	0.84 ± 0.85	15.29 ± 9.96	
Signs of PGP Activity				
IAA production, μg/mL		0		
Gibberellin production, mg/mL		1.26 ± 0.06		
Phosphate mobilization, μg/mL		277.45 ± 46.60		
Ammonium production, μg/mL		0		
Proline production, μg/mL		74.72 ± 2.94		
Salicylic acid production, mg/mL		0		
Siderophore production, %		0		

**Table 2:** Results of two-way analyses of variance examining the effects of stress type, application method, and application timing of *Bacillus subtilis* GMG1288 on wheat growth indicators. Application method refers to three categories: inoculation with or without watering, single watering, and multiple watering. Application timing refers to eight single-application variants at days 0, 14, 21, and 28 (see Section 4.5, “Categorical analysis of application timing”).

Indicator	Factor	df	F	*p*	Partial η^2^
Height	Stress	3	45.60	<0.0001	0.509
	Application method	2	0.54	0.585	0.008
	Application period	3	5.63	0.0017	0.209
	Method × Stress	6	8.74	<0.0001	0.284
	Application period × stress	9	1.74	0.098	0.196
Mass of the leaves	Stress	3	132.56	<0.0001	0.751
	Application method	2	9.58	0.0001	0.127
	Application period	3	5.08	0.0032	0.192
	Method × Stress	6	4.92	0.0001	0.183
	Application period × stress	9	1.45	0.185	0.170
Mass of the root	Stress	3	77.02	<0.0001	0.636
	Application method	2	2.33	0.101	0.034
	Application period	3	3.86	0.0132	0.153
	Method × Stress	6	3.79	0.0016	0.147
	Application period × stress	9	1.48	0.174	0.172
Mass of the plant	Stress	3	145.27	<0.0001	0.768
	Application method	2	7.60	0.0008	0.103
	Application period	3	5.84	0.0014	0.215
	Method × Stress	6	3.12	0.0068	0.124
	Application period × stress	9	1.90	0.068	0.211

## Data Availability

The original contributions presented in this study are included in the article. Further inquiries can be directed to the corresponding authors.
